# Social relationships and health related behaviors among older US adults

**DOI:** 10.1186/1471-2458-14-533

**Published:** 2014-05-30

**Authors:** Richard G Watt, Anja Heilmann, Wael Sabbah, Tim Newton, Tarani Chandola, Jun Aida, Aubrey Sheiham, Michael Marmot, Ichiro Kawachi, Georgios Tsakos

**Affiliations:** 1 Department of Epidemiology and Public Health, University College London, 1-19 Torrington Place, London WC1E 6BT, UK; 2 Dental Institute, Kings College London, London, UK; 3 School of Social Sciences, University of Manchester, Manchester, UK; 4 Department of International and Community Oral Health, Tohoku University Graduate School of Dentistry, Sendai, Japan; 5 Department of Society, Human Development and Health, Harvard School of Public Health, Boston, USA

**Keywords:** Social relationships, Health behaviors, Aging

## Abstract

**Background:**

Health behaviors are a key determinant of health and well-being that are influenced by the nature of the social environment. This study examined associations between social relationships and health-related behaviors among a nationally representative sample of older people.

**Methods:**

We analyzed data from three waves (1999–2004) of the US National Health and Nutrition Examination Survey (NHANES). Participants were 4,014 older Americans aged 60 and over. Log-binomial regression models estimated prevalence ratios (PR) for the associations between social relationships and each of the following health behaviors: alcohol use, smoking, physical activity and dental attendance.

**Results:**

Health-compromising behaviors (smoking, heavy drinking and less frequent dental visits) were related to marital status, while physical activity, a health-promoting behavior, was associated with the size of friendship networks. Smoking was more common among divorced/separated (PR = 2.1; 95% CI: 1.6, 2.7) and widowed (PR = 1.7; 95% CI: 1.3, 2.3) respondents than among those married or cohabiting, after adjusting for socio-demographic background. Heavy drinking was 2.6 times more common among divorced/separated and 1.7 times more common among widowed men compared to married/cohabiting men, while there was no such association among women. For women, heavy drinking was associated with being single (PR = 1.7; 95% CI: 1.0, 2.9). Being widowed was related to a lower prevalence of having visited a dentist compared to being married or living with a partner (PR = 0.92; 95% CI 0.86, 0.99). Those with a larger circle of friends were more likely to be physically active (PR = 1.17; 95% CI:1.06, 1.28 for 5–8 versus less than 5 friends).

**Conclusions:**

Social relationships of older Americans were independently associated with different health-related behaviors, even after adjusting for demographic and socioeconomic determinants. Availability of emotional support did not however mediate these associations. More research is needed to assess if strengthening social relationships would have a significant impact on older people’s health behaviors and ultimately improve their health.

## Background

Health behaviors are a key determinant of population health and well-being. Smoking, excessive alcohol consumption, poor diet and physical inactivity are among the top 10 risk factors for death and disability in high and middle-income countries [1]. Health behaviors are important not only for children and young people, the traditional focus of many health education interventions, but also for adults and older people. Health damaging behaviors such as physical inactivity, poor diet, smoking and heavy alcohol consumption have been associated with an increased risk of disability and death in older people [2-6]. Recently a French cohort study showed the cumulative effect of a combination of three unhealthy behaviors (smoking, low physical activity and low consumption of fruit and vegetables) on disability at the 12 year follow up [7]. Health behaviors also have an important role to play in maintaining good health and promoting successful aging. Sabia and colleagues demonstrated the protective effects of not smoking, moderate alcohol consumption, physical activity and eating fruit and vegetables on a range of positive cognitive, functioning and disease outcomes amongst the sample from the Whitehall II study [8].

Health behaviors are influenced by a complex array of inter related environmental, economic, social, psychological and biological factors [9]. One important determinant of behavior is the nature of the social environment. Most health behaviors are socially patterned in terms of their association with socioeconomic position. A good example of this is smoking where rates are higher in a step wise and consistent fashion as one descends the social hierarchy [10]. The nature, type and quality of social relationships also have a strong influence on patterns of behaviors [11,12]. Most research on social relationships and health behaviors have been conducted amongst children and adolescents [13]. In particular an extensive literature has shown the influence, both positive and negative of peer relationships on adolescent health behaviors [14]. With adults several studies provide evidence that social relationships influence health behaviors [12]. For example, Berkman and Breslow showed in Alameda County that greater overall involvement in formal (e.g. religious organizations) and informal (e.g. friends and family) social ties was associated with more positive health behaviors over a 10 year period [15]. Being married [16], having a family [17] and involvement in religious organizations [18] have all been associated with health promoting behaviors. Social relationships however also carry the potential for encouraging health damaging behaviors such as problem drinking [19]. Individuals with poor social relationships are more likely to smoke, engage in low levels of physical activity and have a poor diet [20-22]. The association between poor social relations and adverse health behaviors appears to be strongest amongst lower socioeconomic groups [23]. Relatively little research has however focused upon the association between social relationships and health behaviors amongst older people [24]. US studies have shown that older people with strong social relationships had better nutrition [25], increased use of mammography [26] and more frequent attendance at a dentist [27]. Older Americans with limited social networks have also been shown to have higher levels of alcohol misuse and physical inactivity [28]. In the UK oral health behaviors were associated with social isolation in a sample of older adults [29].

Patterns of behavior and the nature of social relationships are both dynamic constructs that change across the life course [12]. It is therefore important to explore in detail the association of social relationships with health behaviors at different points in the life course. Previous studies have assessed only certain aspects of social relationships with a limited range of health behaviors. This study therefore assessed the relationship between social networks and social support with four health related behaviors, namely alcohol use, smoking, physical activity and attendance at the dentist amongst a nationally representative sample of older Americans. Further, we tested whether the availability of social support played a mediating role in the associations between social networks and the above behaviors.

## Methods

We analyzed data from the US National Health and Nutrition Examination Survey (NHANES), combining three waves of data that were collected in 1999/2000, 2001/2002 and 2003/2004. NHANES is a rich dataset which contains information on respondent’s general health and self-reported health behaviors, validated instruments measuring social networks and support, as well as a range of socio-demographic background variables. The survey used a stratified, multistage design to obtain a representative sample of the US non-institutionalized population, over-sampling older people, as well as ethnic minority groups. A detailed description of the survey methodology and sampling procedure has been published elsewhere [30,31]. Because the study aim was to examine associations between social relationships and health related behaviors among older people, we restricted the analysis sample to those aged 60 years and over (the same cut-off used in NHANES for oversampling older people), resulting in a sample size of 4,014 participants. The NHANES survey protocol was approved by the National Center for Health Statistics Research Ethics Review Board. All participants provided written informed consent.

### Health-related behaviors

We used four self-reported health behaviors as outcomes: dental visits, smoking, physical activity and alcohol consumption. All outcome variables were dichotomized. Dental visits referred to whether the respondent had visited a dentist at least once during the past two years. The smoking variable distinguished between current smokers (individuals who reported any smoking at the time of the interview), versus non-smokers. Physical activity referred to whether the respondent had engaged in moderate (causing light sweating) or vigorous (causing heavy sweating) physical exercise during the past 30 days (examples of moderate or vigorous activities were brisk walking, cycling, dancing, running, swimming or aerobics classes, but explicitly excluded housework). Heavy drinking was defined according to CDC guidelines, i.e. an average of more than two drinks per day for men and more than one drink per day for women [32]. Respondents were asked about their average alcohol consumption over the past 12 months, and were instructed that one drink equates to 12 fluid ounces (about 350 ml) of beer, 4 ounces (about 120 ml) of wine or 1 ounce (about 30 ml) of liquor.

### Social relationships (social networks and social support)

Social relationships were assessed through social networks and social support variables. Respondents’ social networks were measured via the reported number of close friends, as well as marital status. The variable “How many close friends (relatives or non relatives) do you have?” was divided into (weighted) tertiles (0–4, 5–8, and 9 or more close friends). Marital status was grouped to indicate whether the participants were: married or lived with a partner; widowed; divorced or separated; or single. To assess levels of social support, we derived a binary variable to indicate lack of emotional support. Respondents who replied to the question “Can you count on anyone to provide you with emotional support such as talking over problems or helping you make a difficult decision?” with “No”, or to the question “In the last 12 months, could you have used more emotional support than you received?” with “Yes” were categorized as lacking emotional support. The above measures have been repeatedly used as markers of social relationships in relation to general [33-35], and oral health [36-40].

We also tested potential links with the availability of financial support (“If you need some extra help financially, could you count on anyone to help you?”, possible answers “Yes” or “No”), however the variable was unrelated to any of the outcomes and therefore was not included in the final models.

### Covariates

Our models adjusted for age (in years), sex, ethnicity (White, African American, Mexican American, other Hispanic, and other ethnic group), household income and educational attainment. Household income was measured via the poverty income ratio, which is the ratio between household income and poverty threshold, and is used to account for inflation throughout the years of the survey. Educational attainment was divided into the following three categories: less than high school diploma, high school diploma, and above high school.

### Statistical analysis

Statistical analyses were performed using Stata version 12.1 [41]. After examining bivariate associations between health-related behaviors and explanatory variables, we estimated a series of log-binomial regression models predicting each of the four health behaviors, and report prevalence ratios (PR) and 95% confidence intervals. Log-binomial regression is the preferred method for binary outcomes in cross-sectional and cohort studies when the outcome is common, because in these cases odds ratios obtained from logistic regression models can significantly overestimate prevalence ratios [42]. For each outcome, Model A adjusted for age, sex, and social networks (marital status and number of close friends). Model B additionally adjusted for social support, to test whether the availability of social support mediated associations between respondents’ health behaviors and social networks. Model C then included measures of household income, education and ethnicity, to assess whether the above relationships were independent of socio-economic background characteristics. We also tested whether the strength of the fully adjusted associations varied by sex and income, by including interaction terms between sex/PIR quintiles and each of the social network and support variables.

All analyses were carried out using the NHANES sampling design variables via the Stata *survey* command, to account for the unequal probability of being sampled and the geographical clustering of the data. We derived a six-year sampling weight variable in accordance with NHANES analytic guidelines [30].

## Results

A total of 4,014 participants aged 60 years and older were included in the analysis for whom information was complete. The unweighted mean age of the sample population was 71.7 years (Table 1). In this sample of older Americans, 57% of men and 46% of women had engaged in physical activity over the past 30-days. About 65% of participants had visited a dentist within the past two years, and this was the same for both sexes. Smoking was reported by 14% of men and 11% of women, while 15% of men and 14% of women were classified as heavy drinkers. All examined behaviors appeared to be less prevalent in older age groups. Respondents who were married or living with a partner, had a higher number of close friends and access to sufficient emotional support were more likely to report at least moderate physical activity, to have been seen by a dentist and to be non-smokers in the bivariate analyses. Heavy drinking was also more prevalent among those not living with a partner, however there were no clear associations with the number of close friends and availability of emotional support. The distribution of physical activity, dental visits and smoking followed marked social gradients, with people on higher incomes and a higher level of education being more likely to engage in beneficial behaviors (i.e. physical activity and dental visits) and less likely to be smokers. Heavy drinking was however slightly more prevalent among more affluent respondents and those who were educated beyond high school.

**Table 1:** Health-related behaviors (%), by socio-demographic characteristics and social relationships (weighted)

	**% of N (weighted)**	**Physically active**	**Dental visit less than 2 years ago**	**Current smoker**	**Heavy drinking**
**Social networks/support**					
Marital status					
Married/living with partner	61.9	55.7	70.2	9.9	13.9
Widowed	24.9	41.1	54.6	11.0	11.4
Divorced/separated	10.5	45.5	61.4	25.6	24.1
Single	2.7	38.7	55.8	14.6	22.4
Number of close friends					
0-4	35.8	41.2	56.7	13.6	15.0
5-8	31.6	55.5	69.1	9.9	13.5
9 or more	32.6	56.0	70.1	12.1	15.2
Emotional support					
Sufficient emotional support	81.3	52.8	66.2	11.5	14.4
Lack of emotional support	18.7	40.7	59.8	13.8	15.5
**Socio-demographic characteristics**					
Gender					
Men	44.4	56.8	65.4	13.5	14.9
Women	55.6	45.6	64.7	10.7	14.3
*P-value for chi-square test*		*p < 0.001*	*p = 0.542*	*p < 0.001*	*p = 0.056*
Age group					
60–70	50.7	55.4	67.3	16.4	18.8
71–80	34.0	49.4	64.1	9.0	11.2
81 and older	15.3	37.0	59.3	3.6	8.1
Poverty-income ratio (quartiles)					
Top	25.3	65.9	84.2	8.6	18.0
Second	24.8	57.9	72.6	10.4	14.3
Third	25.1	46.6	62.4	12.5	13.1
Bottom	24.8	32.1	41.1	16.2	13.0
Education					
More than high school	41.5	62.7	81.0	9.7	15.2
High school diploma	29.3	50.1	62.8	12.2	14.9
Less than high school	29.2	33.7	44.4	14.9	13.6
Ethnicity					
White Americans	82.2	52.8	67.2	11.2	13.8
African Americans	7.8	37.0	55.6	16.3	18.2
Mexican Americans	3.1	37.4	53.9	12.6	20.7
Other Hispanic Americans	3.8	37.2	51.2	18.9	17.2
Other	3.1	53.7	57.2	11.5	16.8

### Physical activity

The log-binomial regression models showed that after adjusting for age and sex, a higher number of close friends and being widowed were associated with being physically active, although the latter association was only marginally statistically significant (Model A in Table 2). There was no evidence that the availability of emotional support mediated these associations (Model B in Table 2). After allowing for the influence of income, education and ethnicity, the proportion of those being physically active among respondents who had 5–8 close friends was 1.17 (1.06, 1.28) times, and among those with 9 or more close friends was 1.14 (1.03, 1.27) times higher compared to those who reported to have less than 5 close friends (Model C in Table 2). People who reported access to sufficient levels of emotional support had 1.13 (1.02, 1.26) times higher prevalence of being engaged in physical activity compared to those for whom emotional support was lacking. Marital status was unrelated to physical activity in the fully adjusted model.

**Table 2:** Associations between social relationships and engagement in at least moderate physical activity in the past 30 days

	**Prevalence ratio (95% Confidence interval)**		
	**Model A**	**Model B**	**Model C**
**Social networks/support**			
Marital status			
Married/cohabiting	1.00	1.00	1.00
Widowed	0.88 (0.78, 0.99)*	0.89 (0.79, 1.00)	0.98 (0.88, 1.10)
Divorced/separated	0.86 (0.74, 1.01)	0.88 (0.76, 1.03)	0.98 (0.86, 1.13)
Single	0.78 (0.56, 1.09)	0.79 (0.57, 1.10)	0.84 (0.60, 1.17)
Number of close friends			
0-4	1.00	1.00	1.00
5-8	1.31 (1.19, 1.46)***	1.29 (1.16, 1.43)***	1.17 (1.06, 1.28)**
9 or more	1.28 (1.16, 1.42)***	1.25 (1.12, 1.39)***	1.14 (1.03, 1.27)*
Emotional support			
Lack of emotional support		1.00	1.00
Sufficient emotional support		1.16 (1.05, 1.29)**	1.13 (1.02, 1.26)*
**Covariates**			
Age (years)	0.99 (0.98, 0.99)***	0.99 (0.98, 0.99)***	0.99 (0.99, 1.00)***
Gender			
Male	1.00	1.00	1.00
Female	0.85 (0.79, 0.92)***	0.85 (0.79, 0.91)***	0.88 (0.83, 0.95)**
Poverty-income ratio (cont.)			1.08 (1.04, 1.12)***
Education			
Less than high school			1.00
High school diploma			1.33 (1.13, 1.56)**
More than high school			1.51 (1.30, 1.75)***
Ethnicity (%)			
White Americans			1.00
African Americans			0.87 (0.77, 0.97)*
Mexican Americans			0.93 (0.81, 1.06)
Other Hispanic Americans			0.93 (0.72, 1.21)
Other			1.05 (0.87, 1.27)

***p < 0.001 **p < 0.01 *p < 0.05.

### Dental visits

In the fully adjusted model, being widowed was related to a lower prevalence of having visited a dentist compared to being married or living with a partner (PR = 0.92; 95% CI 0.86, 0.99). There was no association with the number of close friends or the availability of emotional support (Table 3).

**Table 3:** Associations between social relationships and having visited a dentist within the past two years

	**Prevalence ratio (95% Confidence interval)**		
	**Model A**	**Model B**	**Model C**
**Social networks/support**			
Marital status			
Married/cohabiting	1.00	1.00	1.00
Widowed	0.78 (0.71, 0.87)***	0.79 (0.71, 0.87)***	0.92 (0.86, 0.99)*
Divorced/separated	0.90 (0.82, 0.98)*	0.90 (0.82, 0.98)*	1.02 (0.94, 1.10)
Single	0.83 (0.68, 1.00)	0.83 (0.69, 1.00)	0.92 (0.77, 1.09)
Number of close friends			
0-4	1.00	1.00	1.00
5-8	1.20 (1.12, 1.29)***	1.20 (1.12, 1.29)***	1.05 (0.99, 1.12)
9 or more	1.20 (1.11, 1.29)***	1.19 (1.11, 1.28)***	1.05 (0.99, 1.12)
Emotional support			
Lack of emotional support		1.00	1.00
Sufficient emotional support		1.02 (0.93, 1.11)	1.03 (0.96, 1.11)
**Covariates**			
Age (years)	1.00 (0.99, 1.00)	1.00 (0.99, 1.00)	1.00 (1.00, 1.00)
Gender			
Male	1.00	1.00	1.00
Female	1.06 (1.01, 1.12)*	1.06 (1.01, 1.12)*	1.06 (1.01, 1.10)*
Poverty-income ratio (cont.)			1.09 (1.07, 1.11)***
Education			
Less than high school			1.00
High school diploma			1.32 (1.20, 1.44)***
More than high school			1.53 (1.38, 1.69)***
Ethnicity (%)			
White Americans			1.00
African Americans			0.99 (0.94, 1.05)
Mexican Americans			1.01 (0.95, 1.07)
Other Hispanic Americans			1.03 (0.89, 1.18)
Other			0.89 (0.72, 1.11)

***p < 0.001 *p < 0.05.

### Smoking

We found a strong association between smoking and marital status. After taking socio-demographic background variables into account (Model C in Table 4), divorced/separated and widowed participants had significantly higher prevalence of current smoking than respondents who were married or living with a partner, with prevalence ratios of 2.08 (1.59, 2.71) and 1.70 (1.27, 2.27) for the divorced and widowed groups respectively. However, neither the number of close friends, nor the availability of emotional support was independently associated with being a current smoker (Table 4).

**Table 4:** Associations between social relationships and current smoking

	**Prevalence ratio (95% Confidence interval)**		
	**Model A**	**Model B**	**Model C**
**Social networks/support**			
Marital status			
Married/cohabiting	1.00	1.00	1.00
Widowed	2.08 (1.55, 2.79)***	2.08 (1.54, 2.80)***	1.70 (1.27, 2.27)**
Divorced/separated	2.44 (1.89, 3.15)***	2.44 (1.88, 3.17)***	2.08 (1.59, 2.71)***
Single	1.59 (0.90, 2.81)	1.59 (0.90, 2.80)	1.22 (0.67, 2.23)
Number of close friends			
0-4	1.00	1.00	1.00
5-8	0.74 (0.56, 0.98)*	0.74 (0.56, 0.98)*	0.81 (0.61, 1.08)
9 or more	0.94 (0.72, 1.22)	0.93 (0.72, 1.22)	1.02 (0.78, 1.32)
Emotional support			
Lack of emotional support		1.00	1.00
Sufficient emotional support		1.00 (0.80, 1.26)	1.03 (0.82, 1.29)
**Covariates**			
Age (years)	0.92 (0.90, 0.94)***	0.92 (0.90, 0.94)***	0.91 (0.90, 0.93)***
Gender			
Female	1.00	1.00	1.00
Male	1.38 (1.10, 1.74)**	1.38 (1.10, 1.74)**	1.36 (1.07, 1.74)*
Poverty-income ratio (cont.)			0.85 (0.79, 0.91)***
Education			
Less than high school			1.00
High school diploma			0.91 (0.69, 1.22)
More than high school			0.75 (0.59, 0.97)*
Ethnicity (%)			
White Americans			1.00
African Americans			0.89 (0.69, 1.13)
Mexican Americans			0.69 (0.50, 0.95)*
Other Hispanic Americans			1.03 (0.66, 1.60)
Other			0.80 (0.46, 1.40)

***p < 0.001 **p < 0.01 *p < 0.05.

### Alcohol consumption

As with smoking, of the social relationship variables assessed only marital status was independently associated with heavy drinking. However, unlike for the previous behaviors, there were statistically significant interactions by sex: being divorced/separated and being widowed were related to heavy drinking only among men, while being single was associated with heavy drinking only among women (Model C in Table 5). For ease of interpretation, Table 5 presents the associations with marital status for men and women separately. While heavy drinking was no more common among divorced/separated and widowed women than among married or partnered women, drinking heavily was 2.59 (1.89, 3.54) times more common among divorced/separated men than married/cohabiting men. Widowed men had a 1.67 (1.13, 2.47) times higher prevalence of drinking heavily compared to married/cohabiting men. Heavy drinking was however more common among single women than among married/cohabiting women (PR = 1.70; 95% CI 1.01, 2.86). There was a tendency towards a higher prevalence of heavy drinking also among single men, however the result did not reach statistical significance.

**Table 5:** Associations between social relationships and heavy drinking

	**Prevalence ratio (95% Confidence interval)**		
	**Model A**	**Model B**	**Model C**
**Social networks/support**			
Marital status (women)			
Married/cohabiting	1.00	1.00	1.00
Widowed	0.97 (0.66, 1.42)	0.96 (0.69, 1.34)	1.03 (0.73, 1.45)
Divorced/separated	1.21 (0.72, 2.02)	1.15 (0.76, 1.75)	1.24 (0.81, 1.91)
Single	1.83 (0.89, 3.76)	1.58 (0.94, 2.68)	1.70 (1.01, 2.86)*
Marital status (men)			
Married/cohabiting	1.00	1.00	1.00
Widowed	1.59 (1.08, 2.32)*	1.59 (1.09, 2.32)*	1.67 (1.13, 2.47)*
Divorced/separated	2.35 (1.69, 3.26)***	2.37 (1.72, 3.27)***	2.59 (1.89, 3.54)***
Single	1.68 (0.85, 3.32)	1.69 (0.86, 3.35)	1.85 (0.97, 3.54)
Number of close friends			
0-4	1.00	1.00	1.00
5-8	0.91 (0.71, 1.18)	0.91 (0.70, 1.17)	0.90 (0.70, 1.15)
9 or more	1.04 (0.86, 1.27)	1.04 (0.86, 1.26)	1.02 (0.85, 1.24)
Emotional support			
Lack of emotional support		1.00	1.00
Sufficient emotional support		1.03 (0.83, 1.29)	1.03 (0.83, 1.28)
**Covariates**			
Age (years)	0.95 (0.93, 0.97)***	0.95 (0.93, 0.97)***	0.96 (0.94, 0.97)***
Gender			
Female	1.00	1.00	1.00
Male	0.86 (0.70, 1.05)	0.86 (0.70, 1.05)	0.85 (0.69, 1.04)
Poverty-income ratio (cont.)			1.11 (1.03, 1.20)*
Education			
More than high school			1.00
High school diploma			1.07 (0.80, 1.43)
Less than high school			0.96 (0.75, 1.25)
Ethnicity (%)			
White Americans			1.00
African Americans			1.13 (0.90, 1.41)
Mexican Americans			1.45 (1.15, 1.84)**
Other Hispanic Americans			1.26 (0.86, 1.86)
Other			1.05 (0.59, 1.88)

***p < 0.001 **p < 0.01 *p < 0.05.

We also tested for effect modification by income but did not find any significant interactions between the indicators of social relationships and the poverty income ratio for any of the examined outcomes.

## Discussion

This study has shown that social relationships were independently associated with a range of health behaviors in a national sample of older US adults, even after adjusting for the effect of broad demographic and socioeconomic determinants. In particular, being widowed or divorced/separated was significantly associated with being a current smoker, and amongst older men, with being a heavy drinker. Single older women were also more likely to be heavy drinkers. Widowed older people were less likely to attend a dentist. In addition, older people with more close friends more commonly engaged in physical activity. Furthermore, the availability of emotional support did not mediate any of the above associations, but emotional support was associated with physical activity but none of the other behaviors assessed. Access to financial support through social relationships was not related to any of the examined health behaviors.

An extensive body of research has demonstrated the effect of social relationships on health and mortality [43,44]. One of the potential pathways linking social relationships to health is via behavioral factors. However, very few studies have investigated the association between social relationships and health behaviors amongst older people. Frongillo et al. [25] showed that older people with strong social ties had better dietary behavior [25]. In a population of older black Americans, higher levels of social support were associated with attendance at cancer screening services [26]. We are not aware of any other studies that have assessed a combination of health behaviors and their association with both measures of social support and social networks in a sample of older people.

Our findings on the importance of marital status on health behaviors are in accordance with previous reports amongst younger and middle aged adults linking marital dissolution with health compromising behaviors. Longitudinal studies have shown that marital termination, either due to the death of a spouse or divorce, were associated with tobacco and alcohol consumption [45-47]. It is likely that changes in both social support and stress levels are key factors in explaining this link [45]. Smoking and excessive drinking are both related to high levels of stress [48,49] and low levels of social support [50]. Indeed support from a partner may act as a buffer against the harmful effects of stress, and thereby lead to reductions in tobacco and alcohol use [51].

Previous studies have shown that amongst adults, poor social relationships [22] and with older people, limited social networks [28] were both associated with low levels of physical activity. Our results support these findings and highlight the importance of social relationships on physical activity. Older individuals with well-developed and supportive social relationships may be encouraged by their peers and family to adopt and maintain physical activity and may have more information to access local services and amenities.

The associations between social relationships and the three behaviors physical exercise, dental visits and smoking were attenuated after adjusting for socio-economic status, and in the case of dental visits the association was no longer statistically significant. This suggests that those who were poorer and less educated also tended to have smaller friendship networks and were less likely to live with a partner. As expected, there was no attenuation of the results for heavy drinking, which showed a reverse social gradient, i.e. was more common among the more advantaged. Although we observed social gradients in relation to physical exercise, dental visits and smoking, the availability of financial support was not related to any of the examined behavioral outcomes. It is possible that financial support from relatives and friends is given only in the event of an emergency, and is not enough to alleviate the effects of poverty and limited income on patterns of health behaviors.

This study used a large nationally representative sample of US older people, included a range of both health promoting and health damaging behaviors and the detailed analysis controlled for a diverse spectrum of potential confounders. However it is important to acknowledge the limitations of this study. Social support and social networks were only partially assessed due to lack of relevant data, i.e. validated instruments, in NHANES. We only assessed perceived emotional support and did not include any measures of informational and appraisal dimensions of social support. The assessment of social networks covered the size of the networks but did not assess the intensity or quality of social contacts [52]. We also acknowledge that the social network and social support variables could have been categorised in various different ways. For example, it is possible that people with no close friends are very different from people with four close friends. However, having zero friends was very rare in our sample (reported by only 3.7% of all participants). As there is no other cut-off point for this variable that could be conceptually justified, dividing the sample into tertiles was an alternative. Sensitivity analyses using “number of friends” as a continuous variable produced consistent results, i.e. a modest statistically significant association with physical activity but not with the other behaviors. Further to this, we also tested whether dividing the variable “lack of emotional support” into three categories (having someone to provide support plus having received enough support; having someone to provide support but could have used more support; and having no one to provide support) would influence the results, which was not the case. As for the binary variable, the only significant association was with physical activity, with very similar prevalence ratios for the categories “not enough support” and “no support”.

The behavioral outcomes were all assessed through self-report measures which are subject to bias and under reporting. Physical activity in particular is notoriously difficult to assess accurately. NHANES uses its own physical activity questionnaire (PAQ), which although containing an extensive array of questions, cannot be considered to be a validated measure. Our study assessed dental visits as an indicator of health-service use but did not examine the use of other health services, for which associations might be different. Both the social relationship measures and behavioral outcomes were recorded cross-sectionally and did not assess the dynamic nature of these constructs [53]. In the analysis although we adjusted for a range of important covariates, residual confounding may still be an issue. Finally, the cross sectional study design limits any consideration of causal relationships. Indeed it is possible that the association between social relationships and behaviors is due to reverse causation – people who engage in health compromising behaviors may not be able to establish long term stable relationships and older people who smoke and drink heavily might become socially isolated because these behaviors are considered socially undesirable [53].

## Conclusions

In conclusion, this study has shown that social relationships were independently associated with a diverse range of health behaviors amongst a nationally representative US sample of older people. The findings of this study have important implications for public health. Our results provide some evidence that behavioral factors are on the pathway linking social relationships and health. Therefore strengthening and developing social relationships amongst older people should be a priority in health promotion. More interventional studies are however needed to assess if strengthening social relationships would have a significant impact on health behaviors, and ultimately health outcomes amongst older people.

## Competing interests

The authors declare that no competing interests exist.

## Authors’ contributions

RW conceived the study and wrote the paper. AH and WS conducted the analysis of the data. TN, TC, JA, AS, MM, IK and GT contributed to the study design and methodology. GT provided additional statistical support. All authors contributed to the interpretation of the findings, commented on drafts of the paper and approved the final version.

## Pre-publication history

The pre-publication history for this paper can be accessed here:

http://www.biomedcentral.com/1471-2458/14/533/prepub
